# On Valerian in Epilepsy

**Published:** 1850-01

**Authors:** 


*On Valerian in Epilepsy.* By M. Chauffard.—M. Chauffard, an able practitioner at Avignon, adds his testimony to that of so many other writers, concerning the great efficacy of this substance in the various disorders of the nervous system; and in truth in many of the functional disturbances of this it is invaluable. M. Chauffard likewise confirms the opinions of many of the older and some of the modern writers on the materia medica, as to its utility in epilepsy, and he attributes the failures in very many cases in which it has been given to the small quantities employed. To be of use in this formidable affection, very large quantities must be perseveringly taken for some months. In the cases given in illustration, as much as from four to sixteen drachms of the powder were taken in divided doses, daily. Great repugnance is at first felt to both its taste and smell, but this is soon got over, and neither nausea, oppression, nor other disagreeable sensation results; nor does the drug with any certainty produce diuretic or other marked effect. Dr. Chauffard generally premised a bleeding with good effect; and several cases remained under observation for months and years after the cure was effected.—*Brit. and For. Medico-Chirug. Rev., from Rev. Med. Chir.* t. v, pp. 265–72.

[We are almost tired of chronicling remedies for epilepsy, so fallacious do they all sooner or later prove; yet in so terrible an affection, and one in which patients are usually so eager to try every resource, it is satisfactory to have the testimony of so old a practitioner as M. Chauffard, that in some forms of the disease, valerian has, to all appearances, effected a cure.]

